# Posttraumatic stress symptoms among Polish World War II survivors: the role of social acknowledgement

**DOI:** 10.1080/20008198.2018.1423831

**Published:** 2018-01-26

**Authors:** Maja Lis-Turlejska, Szymon Szumiał, Iwona Drapała

**Affiliations:** ^a^ SWPS University of Social Sciences and Humanities, Faculty of Psychology, Warsaw, Poland; ^b^ Caritas Community Self-Help Center, Minsk Mazowiecki, Poland; ^c^ Neuropsychiatric Hospital, Opole, Poland

**Keywords:** Social acknowledgement, World War II, prevalence, PTSD, depression, elderly, reconocimiento social, segunda guerra mundial, prevalencia, TEPT, depresión, ancianidad, 关键词, 社会接纳, 第二次世界大战, 流行率, PTSD, 抑郁, 老年人, • The aim of our study was to measure the associations of social reactions with the levels of PTSD and depression symptoms among WWII survivors in Poland. •The rate of PTSD was very high: 38.3%. •PTSD symptoms and the perceived lack of societal recognition of war trauma were significantly correlated. •The structural equation modelling results also demonstrated the associations of the lack of social acknowledgement with the levels of PTSD and depression symptoms.

## Abstract

**Background**: There is growing evidence of the important role played by socio-interpersonal variables on the maintenance of PTSD. Many World War II survivors in Poland could, as a result of political circumstances during the aftermath of the war, have experienced a lack of social recognition of their war-related trauma.

**Objective**: The main aim of the study was to examine the association between perceived social reactions and the level of posttraumatic stress symptoms (PTSD) and depression.

**Method**: Participants (*N *= 120) were aged 71–97 years (*M *= 82.44; *SD *= 6.14). They completed a WWII trauma-related questionnaire, the Posttraumatic Diagnostic Scale (PDS), the Impact of Events Scale (IES) and Beck’s Depression Inventory (BDI). The Social Acknowledgement Questionnaire (SAQ) was used to measure participants’ perception of others’ acknowledgement and disapproval of their war trauma.

**Results**: The rate of probable PTSD, diagnosed according to DSM-IV, was 38.3%. PTSD symptoms and General Disapproval were significantly correlated for all three PTSD symptom groups (Pearson’s *r* ranged from .25 to .41). The structural equation modelling results also demonstrated the importance of General Disapproval with regard to the level of PTSD symptoms. It explained both the intensity of PTSD symptoms (13.4% of variance) and the level of depression (12.0% of variance).

**Conclusion**: In addition to confirming the high rate of PTSD among WWII survivors in Poland, the results indicate the importance of social reactions to survivors’ traumatic experiences.

## Abstract (es)

**Contexto**: hay una evidencia creciente del importante papel que juegan las variables socio- interpersonales sobre el Trastorno de Estrés Postraumático (TEPT). En Polonia, muchos supervivientes de la segunda guerra mundial pudieron experimentar, como resultado de las circunstancias políticas después de la guerra, una falta de reconocimiento social del trauma que habían desarrollado como resultadi de la guerra.

**Objetivo**: El principal de este estudio fue el examinar la asociación entre las reacciones sociales percibidas y el nivel de síntomas de estrés postraumático(TPET) y depresión.

**Método**: Los participantes (*N*= 120) tenían entre los 71 y 97 años ((*M*=82.44; *SD*=6.14). Completaron un cuestionario relacionado con trauma en la segunda guerra mundial, la escala diagnostica postraumática (PDS), la escala de impacto de eventos (IES) y el inventario de depresión de Beck (BDI). El cuestionario de reconocimiento social (SAQ; Maercker & Müller, 2004) fue utilizado para medir la percepción de los participantes del reconocimiento o rechazo de su trauma bélico por parte de otras personas.

**Resultados**: La tasa de TEPT probable, diagnosticado de acuerdo al DSM IV, fue del 38.3%. Los síntomas de TEPT y la desaprobación general se encontraban correlacionados fuertemente con los tres grupos de síntomas de TEPT (la *r* de Pearson se situaba entre .25 a .41). Los resultados del modelamiento de la ecuación estructural también demostraron la importancia de la desaprobación general en relación con el nivel de síntomas de TEPT. Explicaba tanto la intensidad de los síntomas (13.4% de la varianza) como el nivel de depresión (12.0% de la varianza).

## Abstract (zh)

背景: 关于社会人际变量在 PTSD 的持续中的重要作用有越来越多的证据。在波兰，许多第二次世界大战的幸存者因为战后的政治环境，没有得到对他们战争相关创伤的社会认可。

目标: 研究主要目标在于考察感知到的社会反应和创伤后应激障碍（PTSD）和抑郁症状水平之间关联。

方法: 120名被试年龄范围在71-97岁（M=82.44; SD=6.14）。他们完成了 WWII 创伤相关问卷，创伤后诊断问题（PDS），事件影响问卷（IES）和贝克抑郁量表（BDI）。社会接纳问卷（SAQ; Maercker & Müller, 2004）用于测量被试感知到他人的对他们战争创伤的接纳和否定。

结果: 根据 DSM-IV 的诊断，可能PTSD 的发生率为38.3%。PTSD 症状和总体否定与所有三个 PTSD 症状组显著相关（皮尔逊 r ：0.25 - 0.41）。结构方程模型结果也显示了总体否定对 PTSD 症状水平的重要性。它同时解释了 PTSD 症状的强度（变异的13.4%）和抑郁水平（变异的12%）。

结论: 研究再次证实了第二次世界大战的波兰幸存者中 PTSD 的高流行率，结果提示社会互动对幸存者的创伤体验的重要性。

European studies on mental health among World War II (WWII) survivors – primarily civilians – commenced in the late 1990s (Bramsen & Van Der Ploeg, 1999). Studies primarily concerned with the prevalence of posttraumatic stress disorder (PTSD) have been carried out in Austria (Glück, Tran, & Lueger-Schuster, 2012), Finland (Hautamäki & Coleman, 2001), Germany (e.g. Glaesmer, Gunzelmann, Braehler, Forstmeier, & Maercker, 2010; Kuwert, Spitzer, Träder, Freyberger, & Ermann, 2007), the Netherlands (Bramsen & Van Der Ploeg, 1999) and Norway (Major, 2003). A German study with a nationwide sample of 5033 subjects found that the rate of current PTSD prevalence according to DSM-IV was 4.0% (Glaesmer et al., 2010). In a similar study conducted in the Netherlands, 4.6% of examined subjects fulfilled the criteria of current PTSD, according to DSM-III-R. The highest rate (13%) was found among ‘victims of persecution’ (survivors of German concentration camps, subjects of Jewish origin who survived the war in hiding, survivors of Japanese camps). In other studies conducted in Western European countries in the past decade, the rates of PTSD prevalence ranged from 1.9% in Austria (Glück et al., 2012) to 10.9% in one of the studies in Germany (Kuwert et al., 2007)

Research on analogous groups of WWII survivors in Poland have shown higher levels of PTSD than the studies described above. Lis-Turlejska, Szumiał and Okuniewska (2012) presented a study of 218 persons born from 1929–1945 (then aged 63–78). The prevalence of potential PTSD, according to DSM-IV, was found with the Posttraumatic Diagnostic Scale (PDS) to reach 29.4%. The mean values of symptoms B, C and D were, respectively, 2.08 (*SD *= 1.74), 2.34 (*SD *= 1.98) and 2.40 (*SD *= 1.69). PTSD was predicted by older age, loss of a parent and (on the border of statistical significance) having experienced at least one war-related traumatic experience. In other studies, the rate of PTSD among Polish WWII survivors was found to be 30.09% (Lis-Turlejska, Luszczynska, Plichta, & Benight, 2008) and 32.3% (Lis-Turlejska, Łuszczyńska, & Szumiał, 2016). It is also worth adding that cross-sectional studies of different populations in Poland have shown high levels of PTSD. For example, 19.7% of university-level students met all PTSD criteria as measured with the PDS in a study reported by Dragan, Lis-Turlejska, Popiel, Szumiał and Dragan (2012).

In attempting to explain the reasons for such a large difference between the PTSD levels presented in Western European and Polish studies, several historical, social and political issues must be addressed. The severity of WWII-related stressors in Poland should be considered as one of the reasons for the discrepancy. Poland belongs to the part of Europe that Snyder (2010) called the ‘blood lands’: the area where the regimes of Hitler and Stalin, despite their conflicting goals, interacted to give rise to suffering and bloodshed many times more severe than any witnessed in Western history. One aspect was the scale of human loss: Poland lost about 17% of its pre-war population during WWII – the highest percentage among all countries involved (Davies, 2005; Grabowski, 2009). However, when considering chronic PTSD, risk factors other than those associated with the dose–response relationship should be considered.

In more recent studies and analyses of PTSD, the focus has shifted from individual, intrapsychic risk factors to interpersonal and social ones (Brewin, Andrews, Valentine., & Holloway, 2000; Maercker & Horn, 2013; Ozer, Best, Lipsey, & Weiss, 2003). This perspective has been also stressed by ecological systems models applied in the context of resilience studies on children (Betancourt & Khan, 2008; Reed, Fazel, Jones, Panter-Brick, & Stein, 2011; Ungar, 2012) and in studying now-elderly people who were traumatized as children (Maercker, Hilpert, & Burri, 2016).

Taking into account the wealth of empirical data, Maercker and Horn (2013) presented the PTSD socio-interpersonal model. One of the central constructs in this model is the ‘social acknowledgment of a person as a trauma victim or survivor’. Maercker and Müller (2004) define social acknowledgment as ‘experiencing by the victim a positive response from society that shows appreciation for their exceptional condition and acknowledge their current difficult situation’ (p. 345). In other words, social acknowledgement refers to ‘how a person who has experienced trauma perceives social empathy and understanding by experiencing that the community attributes courage and dignity to survivors because of what they have experienced’ (Maercker et al., 2016, p. 617). ‘Social’ identification means the immediate surroundings of a victim (intimate partner), as well as the significant individuals and groups in a given community and broadly understood society. In a positive case, social acknowledgment includes offering of support to the victims. In contrast, victims may experience negative reactions, such as being ignored, rejected or blamed. Maercker and Müller (2004) constructed a questionnaire to assess the social environment responses toward traumatic event survivors. The Social Acknowledgment as a Victim or Survivor Questionnaire (SAQ) includes both positive (e.g. acknowledgment) and negative (e.g. rejection and disapproval) aspects related to the reactions of a close partner, family, friends and the wider social environment. Self-perceived social acknowledgement has been examined in various populations. Research conducted in Germany, Ingushetia, China and the USA has proven that social acknowledgement is negatively correlated with PTSD symptoms (e.g. Maercker & Müller, 2004; Maercker, Povilonyte, Lianova, & Pöhlmann, 2009; Mueller, Orth, Wang, & Maercker, 2009; Schumm, Koucky & Bartel, 2014).

Perceived social reactions seem especially important in cases of war-related trauma. For example, a lack of social support at the time of homecoming acted as a powerful mediator of trauma and chronic PTSD among American Vietnam War veterans (Fontana & Rosenheck, 1994). Additionally, Koenen, Stellman, Stellman and Sommer (2003) found that high levels of combat exposure and perceived negative community attitudes upon homecoming are the most important predictors of chronic PTSD among war veterans. In a study of veterans seeking PTSD treatment (Schumm, Koucky, & Bartel, 2014), General Disapproval (as measured with the Social Acknowledgment Questionnaire) was positively and significantly related to PTSD, whereas neither Perceived Recognition from closer community members nor Family Disapproval was significantly related to PTSD.

In Poland, the social recognition of war-related trauma has been limited to certain groups only (e.g. Nazi concentration camp survivors), largely due to the political conditions from 1945 to 1989. Most WWII survivors have not ever been recognized as war victims. Large groups of people (e.g. former resistance members who were identified as anti-communists) were prosecuted. Some (approximately one million who were deported from 1940 to 1944 to Siberia from Soviet-occupied areas) were at risk of being prosecuted if they told anyone about their severe traumatization at that time. Moreover, the little attention paid to trauma issues in Polish medicine and psychology as well as the lack of psycho-education could have contributed to difficulties in recognizing and coping with war-related traumatic experiences for many people.

Referring to the Maercker and Horn (2013) PTSD socio-interpersonal model and to the results of previous research, the following research hypotheses were formulated: (1) higher levels of exposure to potentially traumatic events associated with WWII are associated with higher levels of PTSD; (2) higher levels of perceived negative social reactions (i.e. the SAQ General Disapproval score) are associated with higher levels of PTSD symptoms and depression; and (3) lower overall level of social acknowledgment is associated with higher levels of PTSD symptoms.

## 1. Method

### 1.1. Participants and procedure

The participants included 106 females and 70 males aged 71–97 years (*M *= 82.44; *SD *= 6.14). Of these participants, 56 were excluded from the analysis because they either provided incomplete data on the administered questionnaires or were outliers. One hundred and twenty participants (68.2%), including 66 females and 54 males, who were included in the analysis had no missing data and were not outliers on the distributions of all analysed variables. Outliers were defined as those scoring higher or lower than 1.5 times the interquartile range from the 25th to 75th percentiles. According to Pearson’s chi-squared test, the participants who were excluded from the analysis did not differ in terms of sex (χ^2^(1) = .16, *p *> .05), education (χ^2^(5) = 8.89, *p *> .05) or marital status (χ^2^(5) = 7.95, *p *> .05). According to the results of the independent sample *t*-test, these participants also did not differ in terms of age (*t*(171) = −1.95, *p *> .05).

The size of the sample allowed for the detection of effects with a Cohen’s *f* ^2^ value of at least .07 when a statistical power of .80 and a significance level of .05 were assumed. A Cohen’s *f* ^2^ value of at least .07 indicates that the analysis was sensitive to small to medium effect sizes because an *f^2^* of .02 is considered a small effect, and an *f* ^2^ of .15 is considered a medium effect according to Cohen’s guidelines (Cohen, 1988).

Table 1 presents the frequency distributions of the socio-demographical data from the analysed sample.

**Table 1.** Frequency distributions of the sociodemographic characteristics.

Sex	*n*	%
Female	66	55.0
Male	54	45.0
Level of education		
Elementary	29	24.2
Occupational	29	24.2
Secondary	27	22.5
Higher, not completed	12	10.0
Higher, completed	22	18.3
Marital status		
Single	8	6.6
Married	46	38.5
Informal relationship	1	1.1
Divorced	7	5.5
Widow/widower	58	48.4
Total	120	100

*n* = number of participants; % = percentage of the sample

The most frequent level of education was elementary or occupational. In most cases, the participants were widows or widowers. The only difference between the males and females involved the level of General Disapproval (*t*(118) = −2.08, *p *< .05), which was higher among the men (*M *= 10.41; *SD *= 3.96) than the women (*M *= 8.94; *SD *= 3.74).

The participants were recruited from organizations for WWII veterans and deportees to Siberia, nursing homes and cultural centres, and through the personal contacts of the interviewers. The participants completed the questionnaires on their own or with the assistance of the interviewer. The questionnaires were administered at the participants’ homes or the organization’s premises, or the participants took the questionnaires home and they were collected a few days later. The interviewers were graduate clinical psychology students.

### 1.2. Measures

A list of events associated with WWII (Lis-Turlejska et al., 2016) included 18 potentially traumatic events that the participants directly experienced (e.g. torture, rape, imprisonment in Nazi concentration camps, loss of one’s mother, bombing, extreme hunger, killing a person) and six events that they witnessed (shooting of a person, execution, rape or any other sexual violence, heavy beating of a person, assault, persecution of Jews). The respondents were asked to answer each item in a yes/no format. The sum of the ‘yes’ answers was considered as a result, i.e. the number of traumatic events.

The Social Acknowledgment Questionnaire (SAQ; Maercker & Müller, 2004; Polish adaptation: I. Drapała, M. Lis-Turlejska) is a self-report scale with 16 items that assesses the degree to which an affected person perceives that his or her experience is acknowledged by his or her social network following a traumatic event. The participants’ responses are rated on a 4-point Likert scale ranging from *totally disagree* to *totally agree*. The SAQ refers to positive (e.g. recognition) and negative (e.g. rejection, disapproval) aspects of perceived social reactions. The scale consists of three subscales: General Disapproval (refers to general society, e.g. ‘Somehow I am no longer a normal member of society since the incident’); Recognition as a Victim (which refers to acquaintances, friends and locally important public figures, e.g. ‘My friends showed sympathy for what happened to me’); and Family Disapproval (e.g. ‘My family feels that they have to protect me’). Higher scores on the subscales of General Disapproval and Family Disapproval represent negative social responses, and higher scores on the Recognition subscale indicate perceived positive responses to trauma. The original version has good psychometric features. The reliabilities of the subscores and total score are satisfactory, with Cronbach’s *α* values ranging from .79 to .87 (Maercker & Müller, 2004). The Cronbach’s *α* values for the SAQ subscales and total scores from the current study are presented in Table 6, and they ranged from .61 to .80.

**Table 2.** Frequency distributions of the number and types of traumatic events in the sample.

Number of traumatic events	*n*	%
0	3	2.5
1	4	3.3
2–5	31	25.8
6–10	54	45.0
11–19	28	23.3
Total	120	100
Type of traumatic event		
Lost one’s mother	18	15.0
Lost one’s father	36	30.0
Lost one’s close relative	50	41.7
Was in combat	11	9.2
Was in resistance	13	10.8
Was wounded	22	18.3
Killed someone	15	12.5
Was tortured	8	6.7
Was imprisoned in a Nazi concentration camp	9	7.5
Was imprisoned in a Soviet camp	6	5.0
Was in a ghetto	2	1.7
Was in Warsaw during the Warsaw Uprising	33	27.5
Participated in the Warsaw Uprising	22	18.3
Experienced rape or other form of sexual abuse	9	7.5
Survived bombing	98	81.7
Had to remain in hiding	64	53.3
Hid Jews	18	15.0
Was forcedly deported to Siberia	28	23.3
Was in forced labour in Germany	13	10.8
Experienced health- or life-threatening cold	61	50.8
Experienced life-threatening hunger	75	62.5
Witnessed combat	75	62.5
Witnessed somebody being shot	65	54.2
Witnessed execution or murder	49	40.8
Witnessed rape or other form of sexual abuse	28	23.3
Witnessed somebody being heavily beaten	47	39.2
Witnessed assault or persecution of Jews	43	35.8

*n* = number of participants; % = percentage of the sample

**Table 3.** Frequency distribution of the level of depression in the sample.

Level of depression	*n*	%
None or minimal (<10)	26	21.7
Mild to moderate (10–18)	51	42.5
Moderate to severe (19–29)	39	32.5
Severe (30–63)	4	3.3
Total	120	100

*n* = number of participants; % = percentage of the sample

**Table 4.** Results of confirmatory factor analysis applied to SAQ: factor loadings with standard errors.

Item	General disapproval	Recognition	Family disapproval	*SE*
7	.84			
5	.83			.10
4	.69			.09
2	.36			.09
1	.59			.10
15		.32		
14		.73		.80
13		.80		.79
12		.93		1.06
3		.46		.58
16		.34		.28
10			.81	.14
9			.26	.12
8			.83	.08
6			.81	
Second-order factor loadings				
Total score – General Disapproval			1.07	
Total score – Recognition			.58	.05
Total score – Family Disapproval			.66	.10

*SE* = standard error

**Table 5.** Descriptive statistics for the interval scales.

Questionnaires	Variables	*M*	*SD*	min	max	α
PDS	Intensity of PTSD B symptoms	4.28	3.10	0	15	0.82
	Intensity of PTSD C symptoms	4.29	4.09	0	17	0.80
	Intensity of PTSD D symptoms	4.67	3.46	0	14	0.77
	Intensity of PTSD symptoms total	13.24	9.32	0	44	0.91
IES	Intrusion	16.75	9.58	0	35	0.89
	Avoidance	15.50	5.76	0	36	0.84
	IES total score	32.25	17.46	0	67	0.91
BDI	Depression	15.72	8.22	0	47	0.88
SAQ	General Disapproval	9.60	3.89	0	18	0.80
	Recognition	10.14	3.81	0	21	0.80
	Family Disapproval	6.93	3.86	0	18	0.79
	SAQ total score	28.53	6.33	12	45	0.61

*M* = mean; *SD* = standard deviation; min *=* minimum; max = maximum; α = Cronbach’s α reliability coefficient

**Table 6.** Pearson correlation coefficients between analysed interval variables.

	2.	3.	4.	5.	6.	7.	8.	9.	10.	11.	12.	13.
1. No. of traumatic events	.19*	.09	.22**	.19*	.36**	.10	.26**	.15*	−.01	−.10	.14	.11
2. PTSD symptoms criterion B	-	.64**	.67**	.87**	.55**	.42**	.54**	.41**	.05	.17*	.08	.41**
3. PTSD symptoms criterion C		-	.62**	.89**	.29**	.30**	.33**	.31**	.01	.26**	.01	.30**
4. PTSD symptoms criterion D			-	.87**	.44**	.30**	.41**	.25**	.03	.04	.07	.39**
5. PTSD symptoms total					.48**	.38**	.48**	.37**	.03	.19*	.06	.41**
6. Intrusion					-	.63**	.90**	.12	−.13	−.23**	.10	.27**
7. Avoidance						-	.90**	.17*	.13	−.04	.18*	.24**
8. IES total score							-	.16*	.01	−.15	.15*	.28**
9. General Disapproval								-	.39**	.53**	.09	.34**
10. Recognition									-	.39**	.63**	.18*
11. Family Disapproval										-	.10	.15
12. SAQ total score											-	.02
13. Depression												-

* p < .05; ** p < .01

The Posttraumatic Diagnostic Scale (PDS; Foa, Cashman, Jaycox, & Perry, 1997; Polish adaptation: Dragan et al., 2012) is a self-report scale that was designed to measure the presence and severity of PTSD symptoms in addition to detecting persons with diagnosable PTSD. The PDS refers to the main diagnostic categories of the DSM-IV, i.e. re-experiencing (5 items), avoidance/numbing (7 items) and arousal (5 items). Participants’ responses on the 4-point scale ranged from 0 to 3. The original list of traumatic events was replaced with a list of 24 WWII-related potentially traumatic events. The original version of the scale is characterized by high internal consistency (*r* = .92), good test-retest reliability (*r* = .74) for the diagnosis of PTSD and *r* = .83 for the intensity of symptoms (Foa et al., 1997). In the current study, the Cronbach’s α for the intensity of PTSD symptoms total score was .91.

The Impact of Event Scale (IES; Horowitz, Wilner, & Alvarez, 1979; Polish adaptation: M. Lis-Turlejska & A. Łuszczyńska) allows for measurement of the posttraumatic symptoms of intrusions and avoidance. The scale consists of 15 items that describe symptoms belonging to both categories. Participants report their responses on a 4-point Likert scale. In the current study scoring was: 0, 1, 3, 5. IES has been one of most popular measures used in the PTSD studies internationally. In the current study, the Cronbach’s *α* for the IES total score was .91.

The Beck Depression Inventory (BDI; Beck, Ward, Mendelson, Mock, & Erbaugh, 1961; translated by M. Lewicka & J. Czapiński) is a 21-item multiple-choice self-report inventory that is widely used to measure the presence and degree of depression. It includes both cognitive and somatic symptoms of depression. The intensity of each symptom is rated from 0 to 3. Zero indicates that the symptom is not present, whereas three indicates the most extreme level of the symptom. The internal consistency of the BDI ranges from 0.73 to 0.92 with a mean of .86 (Beck, Steer, & Garbin, 1988). The Cronbach’s *α* for the BDI in the current study was .88.

#### 1.3. Data analysis

The participants were 378 selected for data analysis with the use of boxplots that enabled the identification of outliers. The first step of the analysis was to compute the frequency distributions of the participants’ genders and education levels, the numbers of war-related potentially traumatic events experienced, the percentages of probable PTSD, the levels of depression and the types of war-related potentially traumatic events experienced.

The factorial structure of the SAQ was verified by confirmatory factor analysis, and the necessary corrections were performed. Descriptive statistics were computed for all interval scales along with Cronbach’s α reliability coefficients. Correlation analyses were performed to identify statistically significant associations between all interval variables.

Path analysis was conducted using the maximum likelihood method to verify the hypothesized model considering the processes involving the number of traumatic events, social acknowledgement and PTSD and depression symptoms. To verify the model, CFI, TLI and RMSEA fit indexes were used. Computations were performed using IBM SPSS Statistics 24.0 and IBM SPSS AMOS software (IBM, 2016, New York, USA).

## 2. Results

### 2.1. Prevalences of war-related potentially traumatic events, probable PTSD and depression

Table 2 presents the frequency distributions of the number of potentially traumatic events and the types of war-related traumatic events in the sample. The number of potentially traumatic events ranged from 0 to 19, with a mean value of 7.71 and a standard deviation equal to 4.00.

Forty-six participants (38.3%) met the DSM-IV criteria for a PTSD diagnosis. Fifty-four participants (45.0%) had IES total scores above or equal to the cut-off value of 35, which indicates a level of PTSD symptoms characteristic of PTSD diagnosis. In the current study, a cut-off of 35 was applied, as it had the highest predictive value (.88) and lowest false discovery and false negative rates (Joseph, 2000; see also Haagsma et al., 2012).

Table 3 presents the frequency distribution for depression levels in the sample according to the guideline cut-off scores of Beck et al. (1988). Most of the participants had mild to moderate depression.

### 2.2. CFA

To test the SAQ factor structure, confirmatory factor analysis was conducted. Computations were performed using the maximum likelihood method. The 3-factor structure presented by Maercker and Müller (2004) led to results with values slightly below expectations. The model significantly deviated from the observed data, i.e. χ*^2^*(89, *N *= 120) = 119.35, *p *= .018, *CFI* = .96, *TLI *= .95 and *RMSEA *= .05 [90% *CI *= .02, .08].

The reason for this deviation was that item 11 (‘My family showed a lot of understanding towards me after the incident’) was not significantly related to the Family Disapproval scale (*Beta *= .17, *p *> 0.05). This item was removed from the scale. After the correction, the model fit was acceptable, i.e. χ*^2^*(75, *N* = 120) = 91.19, *p *= .098, *CFI* = .98, *TLI* = .97 and *RMSEA* = .04 [90% *CI* = .01, .07]. Table 4 presents the factor loadings with the standard errors.

### 2.3. Descriptive statistics for the interval scales

Table 5 presents the descriptive statistics for the interval scales and Cronbach’s α reliability coefficients. Nearly all of the reliability coefficients were satisfactory, with the exception of the SAQ total score for which the α coefficient was moderate.

### 2.4. Correlation analysis

Table 6 presents the Pearson correlation coefficients between the analysed interval variables.

The number of potentially traumatic events was positively correlated with the intensity of PTSD symptoms (criteria B and D). The number of potentially traumatic events was also positively correlated with General Disapproval.

The intensities of PTSD symptoms included in criteria B, C and D were significantly positively correlated with each other. All of these values were positively correlated with General Disapproval. The intensities of PTSD symptoms included in criteria B and C were positively correlated with Family Disapproval. The intrusion, avoidance and IES total scores were also correlated with each other and with the intensities of PTSD symptoms included in all of the criteria, as measured with the PDS.

The avoidance and IES total scores were positively correlated with the General Disapproval and the SAQ total scores. Intrusion was negatively correlated with Family Disapproval.

### 2.5. Path analysis

**Figure 1.**
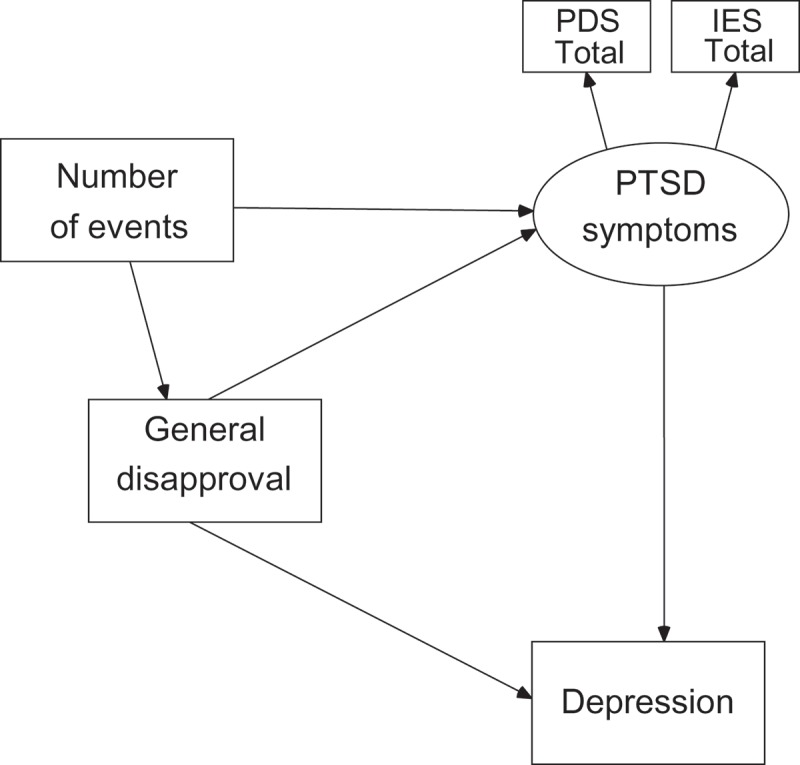
Associations between number of traumatic events, general disapproval, PTSD symptoms and depression: entry model.

The associations between the number of potentially traumatic events, General Disapproval, PTSD symptoms and depression were tested by structural equation modelling. Of the four variables that concerned the social acknowledgement concept (i.e. General Disapproval, Recognition, Family Disapproval, SAQ total score), General Disapproval was included in the model because it was significantly correlated with the number of potentially traumatic events and PTSD symptoms; thus, it could mediate between the two. The entry model is presented in Figure 1.

**Figure 2.**
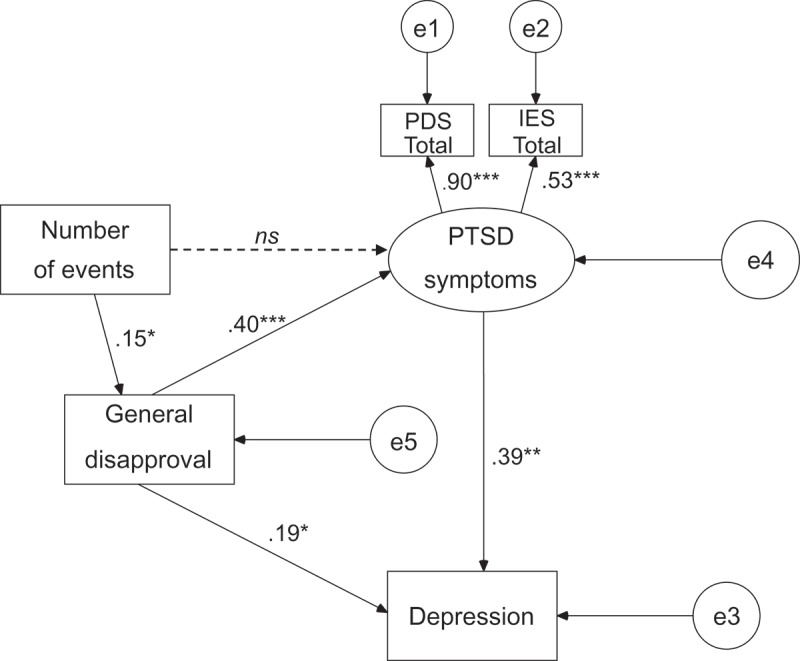
Associations between number of traumatic events, general disapproval, PTSD symptoms and depression: final model. * p < .05; ** p < .01; *** p < .001; ns = not significant

Figure 2 presents the final model after the modifications. The fit of the model was acceptable, i.e. χ*^2^*(4, *N *= 120) = 8.14, *p *> 0.05, *CFI *= .95, *TLI *= .95 and *RMSEA *= .04 [90% *CI* = .01 ÷ .19]. The entry model (Figure 1) assumed a direct association between the number of potentially traumatic events and PTSD symptoms. However, this path was not statistically significant when the level of General Disapproval was taken into account, so it was excluded from the final model. General Disapproval appeared to be explained by the number of potentially traumatic events and, in turn, General Disapproval explained the severity of PTSD symptoms and the level of depression. A greater number of potentially traumatic events was associated with a higher level of General Disapproval which, in turn, was associated with more severe PTSD symptoms and depression. The level of depression was explained partly by General Disapproval and partly by PTSD symptoms.

General Disapproval explained 16% of the severity of PTSD symptoms. General Disapproval and PTSD symptoms both explained 24% of the variance in depression.

## 3. Discussion

The main goal of the study was to estimate the associations between perceived social reactions to WWII trauma-related experiences and symptoms of PTSD and depression among people who had survived the war. The other goals included estimating the PTSD and depression levels.

Our findings showed statistically significant (*p *< .01) positive correlations between the intensity of PTSD symptoms included in criteria B, C and D and the PDS total score with General Disapproval. The intensity of PTSD symptoms included in criterion B and C and the PDS total score also correlated positively with Family Disapproval. There were no correlations between any measure of PTSD and Recognition.

The SEM results also showed the importance of General Disapproval in relation to the level of PTSD symptoms. General Disapproval explained both the severity of PTSD symptoms and the level of depression. However, it was also related to the number of traumatic events; a higher number of traumatic events was related to a higher level of General Disapproval, which in turn was related to more severe PTSD symptoms and depression. Moreover, the level of depression was partly explained by General Disapproval.

Similar results showing that General Disapproval was positively and significantly related to PTSD have been presented by Mueller, Moergeli and Maercker (2008) in a study of crime victims. Schumm et al. (2014), who performed structural equation modelling of the results of their study of US military veterans seeking PTSD treatment, found that General Disapproval was positively and significantly related to PTSD, whereas both Recognition and Family Disapproval were not. The results of their study also showed that General Disapproval was the only SAQ factor that was significantly related to both PTSD and depression.

The lack of correlation between Recognition and the level of PTSD symptoms is contrary to the assumptions of Maercker and Müller (2004) regarding the importance of social acknowledgment from different circles – an intimate partner, family members, the community and general society. However, regarding the scale of traumatization during WWII in Poland (e.g. Davies, 2005; Grabowski, 2009), we can assume that almost everybody was affected and that there were not enough psychological resources available to offer help and acknowledgement for severely distressed people. It can be understood that people did not have enough resources to support each other, as everybody needed support. Family Disapproval correlates with PTSD symptoms, as expected. The *r* coefficients, however, are lower than the correlations with General Disapproval.

The mean number of potentially traumatic events associated with the war was: *M *= 7.71 (*SD* = 4). Thirty percent of the subjects lost their father, and 41.7% lost a close relative. The PTSD prevalence in the current study was 38.3%, which is even higher than those reported in previous studies (Lis-Turlejska et al., 2016, 2012). The increase observed in the PTSD rate was attributed to a subgroup (*n *= 28) of participants deported to Siberia from the years 1941 to 1944.

The PTSD rate of that value seems very high considering the results of the studies on WWII survivors done in several Western European countries (e.g. Bramsen & Van Der Ploeg, 1999; Glaesmer et al., 2010; Glück et al., 2012). A similar PTSD rate (38.6%) was reported among a group of individuals undergoing medical treatment in a study of the victims of war in the former Yugoslavia three years after the war (Rosner, Powell, & Butollo, 2003). Additionally, the level of depression in the studied group was high. The mean BDI score in the current study was 15.72 (*SD *= 8.22). Further, in a study of torture survivors undergoing individual treatment at rehabilitation centres in five different countries, the mean BDI score was 14.2 (*SD *= 9.8), and the PTSD prevalence in that group was 40% (McColl et al., 2010).

While considering the PTSD level observed in the current study, it is worth considering the impact of the age of the studied group. Cook (2001) points to several reasons why symptoms of PTSD can increase with age. For instance, role changes and functional loses may make coping with memories of earlier trauma more challenging for the older adult. Stressors related with elderly include problems contributing to functional difficulties. Such stressors include retirement, increased health problems, decreased sensory abilities, reduced income, loss of loved ones, decreased social support and cognitive impairment. However, adaptation and resilience developed over a lifetime can provide a reservoir of coping resources upon which to draw. Evidence indicates that the PTSD prevalence is probably lower in older men than in younger men. Schnurr, Spiro, Vielhauer, Findler and Hamblen (2002) assessed lifetime trauma exposure and PTSD among WWII and Korean Conflict veterans. Despite a high prevalence of trauma exposure, the veterans’ symptom levels were relatively low. Few of the men met the criteria for current or lifetime PTSD. According to the authors, their findings call for wider investigation of trauma and its consequences in older populations.

The results of our study shed some light on the reasons for the high PTSD levels among Polish WWII survivors observed in both the current study and previous studies (Lis-Turlejska et al., 2016, 2012). General Disapproval appeared to be the most important factor associated with the level of PTSD, as well as with depression.

While looking at the history after WWII in Poland, we can see that the WWII survivors have experienced a lack of social acknowledgement of the war trauma and societal disapproval. The experiences of the Poles deported to Siberia from the area under the Soviet Union’s occupation from 1941 to 1944 (their number is estimated to be between 800,000 and 1.5 million; Ciesielski, 1999) can serve as an example. Many people died because of freezing temperatures, hunger and work exhaustion. Those who returned to Poland after the end of the war had lost their homes and were sent to new locations. Particularly during the so-called ‘Stalinist era’ between 1947 and 1956, repression was widespread and included the imprisonment and torture of people who were accused of being against the communist system. For the people who had been sent to Siberia, this repression meant that they could not talk about their suffering and losses during the war. The rates of PTSD among individuals deported to Siberia, as estimated in several studies, appear to be very high. In her study, Jackowska (2005) has estimated the rate of PTSD to be more than 50%. Additionally, Paszko (2016) studied a group of deportees to Siberia using the PDS and IES and found that the prevalence of PTSD according to DSM-IV criteria was 50%.

Attempting to explain possible causes of the high prevalence of PTSD among Polish WWII survivors appears to be an important task. The severity of WWII-related stressors in Poland should be considered one of the reasons. However, the present study showed the role of lack of general social acknowledgement as a risk factor for PTSD. Providing psycho-education and psychological intervention focused on the processing of war trauma among WWII survivors, as did Forstmeier, Maercker, Van Der Hal-Van Raalte and Auerbach (2014) and Knaevelsrud et al. (2014), seems to be important.

Several limitations of this study should be considered. The first one was using a convenience sample of persons born before 1945. Possibly the findings of the study should be replicated on the representative study of persons who survived WWII. However, this was overcome to some extent because the participants were approached in several regions of the country – both small towns as well as the capital and in different organizations as well as individually. Lack of a screening procedure regarding the cognitive abilities of the participants was the other limitation. To avoid too much missing data in the further study regarding the old age of the population studied, the screening procedure for cognitive abilities enabling understanding and completing measurement tools should be applied. Taking into consideration the massive scale of traumatization during WWII in Poland and the influence of a totalitarian political system after the war, it would be difficult or even impossible to use the control group design.
